# Magnolol, a natural compound, induces apoptosis of SGC-7901 human gastric adenocarcinoma cells via the mitochondrial and PI3K/Akt signaling pathways

**DOI:** 10.3892/ijo.2011.1277

**Published:** 2011-11-30

**Authors:** AZHAR RASUL, BO YU, MUHAMMAD KHAN, KUN ZHANG, FURHAN IQBAL, TONGHUI MA, HONG YANG

**Affiliations:** 1 Central Research Laboratory, Jilin University Bethune Second Hospital, Changchun 130041; 2 School of Life Sciences, Liaoning Normal University, Dalian 116029, P.R. China; 3 Institute of Pure and Applied Biology, Bahauddin Zakariya University, Multan, Pakistan

**Keywords:** Magnolol, natural compounds, apoptosis, S-phase arrest, SGC-7901 cells

## Abstract

Gastric cancer is the fourth most commonly diagnosed cancer with the second highest mortality rate worldwide. Surgery, chemotherapy and radiation therapy are generally used for the treatment of stomach cancer but only limited clinical response is shown by these therapies and still no effectual therapy for advanced gastric adenocarcinoma patients is available. Therefore, there is a need to identify other therapeutic agents against this life-threatening disease. Plants are considered as one of the most important sources for the development of anticancer drugs. Magnolol, a natural compound possesses anticancer properties. However, effects of Magnolol on human gastric cancer remain unexplored. The effects of Magnolol on the viability of SGC-7901 cells were determined by the MTT assay. Apoptosis, mitochondrial membrane potential and cell cycle were evaluated by flow cytometry. Protein expression of Bcl-2, Bax, caspase-3 and PI3K/Akt was analysed by Western blotting. Magnolol induced morphological changes in SGC-7901 cells and its cytotoxic effects were linked with DNA damage, apoptosis and S-phase arrest in a dose-dependent manner. Magnolol triggered the mitochondrial-mediated apoptosis pathway as shown by an increased ratio of Bax/Bcl-2, dissipation of mitochondrial membrane potential (ΔΨm), and sequential activation of caspase-3 and inhibition of PI3K/Akt. Additionally, Magnolol induced autophagy in SGC-7901 cells at high concentration but was not involved in cell death. Magnolol-induced apoptosis of SGC-7901 cells involves mitochondria and PI3K/Akt-dependent pathways. These findings provide evidence that Magnolol is a promising natural compound for the treatment of gastric cancer and may represent a candidate for *in vivo* studies of monotherapies or combination antitumor therapies.

## Introduction

Gastric cancer is the second most common cause of cancer-related death worldwide and approximately 800,000 people die each year of this malignancy. So far it is the fourth most frequently diagnosed cancer as each year more than one million patients are annually diagnosed with gastric cancer (1,2). The incidence of stomach cancer varies geographically, with a much higher prevalence in Eastern countries than in the Western ones (3). In 2005, the incidence of gastric cancer (0.3 million deaths and 0.4 million new cases) ranked third among the most common cancers in China (4). Although surgery remains the gold standard for the treatment of stomach cancer but the limitations is that it is diagnosed at an advanced stage. The 5-year survival rate of patients with advanced gastric cancer for surgical treatment is less than 40%. The effectiveness of chemotherapy and/or radiation therapy, in addition to surgery, has been actively studied over the last few decades. Unfortunately, only a little clinical response is generally shown by chemotherapy or radiation therapy and survival rate is also very poor (5). There is no effective therapy for patients with advanced gastric adenocarcinoma. Therefore, to identify new therapeutic agents against gastric cancer is the critical requirement to improve health and survival chances of the patients.

The purpose of this study was to find new anticancer agents to cure gastric cancer and for this purpose we performed screening to find novel compounds for treatment of gastric cancer. During the screening program, to evaluate the potential chemopreventive effect of natural compounds, we screened 400 single compounds against human gastric adenocarcinoma SGC-7901 cells. Magnolol was one of them that showed antiproliferative effects against gastric adenocarcinoma SGC-7901 cells. Several studies have documented that many compounds, originally isolated from plants such as the paclitaxel, camptothecin, vinca alkaloids, and etoposide are used in cancer chemotherapy. Plants are considered as one of the most important sources for the development of innovative anti-cancer drugs (6–8).

Magnolol, a natural compound has been reported to have wide spectrum of biological effects including antioxidant (9–11), antithrombotic (12), antimicrobial (13), anti-allergic (14), antifungal (15), anti-inflammatory (16), and xanthine oxidase inhibition (17). Moreover, Magnolol induced antiproliferative effects in wide variety of tumor cells including melanoma cells (18–21), colon cancer cells (22–25), prostate cancer cells (26,27), human glioblastoma cancer cells (28,29), liver cancer cells (24,25), lung cancer cells (30,31), leukemic cells (14), cervical cancer (32), ovarian cancer cells (32), thyroid carcinoma cells (33), human fibrosarcoma HT-1080 (34), and human urinary bladder cancer 5637 cells (35,36). Several researchers reported that Magnolol-induced cell death involve apoptosis while Li *et al* (30), reported that Magnolol-induced death occurs via autophagy but not apoptosis. Accumulated data indicate that mechanism of Magnolol-induced cell death varies with cell type. However, effects of Magnolol and its mechanism on human gastric adenocarcinoma cells remain unexplored. Therefore, the present study was conducted to explore the effects of Magnolol on the proliferation of human gastric adenocarcinoma SGC-7901 cells and its mechanism. Moreover, to examine whether Magnolol-induced cell death occurs via apoptosis, autophagy, or both. Results indicated that Magnolol effectively inhibited the proliferation of SGC-7901 cells through arresting the cell cycle at S-phase and induction of apoptosis which is regulated by activation of caspase-3, down-regulation of Bcl-2, and up-regulation of Bax.

## Materials and methods

### Chemicals and reagents

Magnolol was purchased from the National Institute for the Control of Pharmaceutical and Biological Products (Beijing, China). Fetal bovine serum was purchased from Hangzhou Sijiqing Biological Engineering Materials Co., Ltd. DMEM, MTT [3′-(4,5-dimethylthiazol-2-yl)-2,5-diphenyl tetrazolium bromide], propidium iodide (PI), and dimethyl sulfoxide (DMSO) were purchased from Sigma Chemical Co. (St. Louis, MO, USA). Annexin V-FITC Apoptosis Detection Kit was purchased from Beyotime Institute of Biotechnology (Shanghai, China). Rabbit polyclonal anti-human Bcl-2, anti-human Bax and cleaved caspase-3 antibodies were purchased from Wuhan Boster Biological Technology Co., Ltd., Phospho-Akt (Ser-473), PI3K, and Akt antibodies were purchased from Cell Signalling Technology (Beverly, MA, USA). Mouse anti-β-actin and anti-rabbit antibodies were purchased from Santa Cruz Biotechnology. Ponceou and cell lysis buffer for Western blotting and IP were purchased from Bio SS Beijing. Rhodamine 123 was purchased from Eugene Co. (OR, USA).

### Cell culture

Human gastric adenocarcinoma SGC-7901 cells were cultured and maintained in DMEM supplemented with 10% fetal bovine serum (FBS), 100 μm/l penicillin, and 100 μg/ml streptomycin at 37°C in a humidified atmosphere with 5% carbon dioxide and 95% air. Cells were cultured in a 10-cm culture dish and were allowed to grow to ~60–80% confluence before experimentation.

### Cell proliferation assay

The effect of Magnolol on the viability of cells was examined by the MTT assay. SGC-7901 cells were sub-cultured in 96-well plates and were allowed to adhere overnight. Next day, cells were treated with various concentrations of Magnolol (0, 10, 30, 50, 100, 200 and 300 μM) for 48 h. After incubation, 10 μl of MTT (5 mg/ml in phosphate buffered saline) was added to each well and incubated further for 4 h. Medium was aspirated carefully and 150 μl of DMSO was added to each well. The absorbance was measured on the Microplate Reader (ELX 800, Bio-Tek Instruments, Inc.) at the wavelength of 570 nm. The effects of Magnolol were determined on viability of cells and inhibition ratio (I%) was calculated using the following equation (37):

$$I\%=[A_{570}\;(control)-A_{570}\;(treated)]/A_{570}\;(control)\times 100$$

### Determination of apoptosis by flow cytometry

Apoptosis was determined through Annexin V-FITC Apoptosis Detection Kit. SGC-7901 cells were seeded in 6-well plates and were incubated overnight and then treated with 40, 60, and 80 μM of Magnolol, respectively, for 48 h. Cells were harvested by trypsinization, washed with pre-chilled PBS (4°C) and centrifuged at 1000 rpm for 5 min. The cell pellet was resuspended in 195 μl of binding buffer and incubated with 5 μl Annexin V-FITC in the dark at room temperature for 10 min. Cells were centrifuged, washed with PBS, and re-suspended in 195 μl of binding buffer containing 10 μl PI solution in the dark and were then analyzed by flow cytometry (Beckman FC400 MPL, USA).

### Cell cycle analysis

The distribution of cells in different phases of cell cycle after exposure of Magnolol was analyzed with flow cytometry. Briefly, SGC-7901 cells were harvested and washed with PBS after exposure of 40, 60 and 80 μM of Magnolol with control group for 48 h. The cells were fixed with 70% cold ethanol at −20°C overnight and then stained with PI solution consisting of 1 mg/ml PI and RNase A. The fluorescence-activated cells were sorted in the flow cytometry, and the data were analyzed using CellQuest analysis software.

### Flow cytometric analysis of mitochondrial membrane potential

Mitochondrial transmembrane potential was assessed by Rho-123 staining as we described previously (37). Briefly, cells were incubated without (control) and with (40, 60 and 80 μM) Magnolol for 48 h. After the incubation, cells were collected, cell pellets were washed twice with ice-cold PBS and then incubated with Rho-123 (1 μM) at 37°C for 20 min. Stained cells were washed twice with PBS, resuspended in 0.5 ml of PBS followed by flow cytometric analysis. The fluorescence of treated cells was compared with control group.

### Immunoblotting

To elucidate the mechanism of the apoptotic effect of Magnolol, we analyzed the apoptosis-related proteins in SGC-7901 cells. After incubation of cells without (control) and with (40, 60, and 80 μM) Magnolol for 48 h, cells were harvested, washed twice with PBS, and cell lysates were prepared using lysis buffer. Protein estimation was done using NanoDrop 1000 spectrophotometer (Thermo Scientific, USA). An equal amount of protein lysates of cells were subjected to SDS-PAGE followed by Western blotting. The membranes were soaked in blocking buffer (5% skimmed milk) for 2 h in TBST at room temperature. To probe for Bcl-2, Bax, cleaved caspase-3, phospho-Akt, pPI3K, Akt, and β-actin, membranes were incubated overnight at 4°C with relevant antibodies, followed by appropriate HRP conjugated secondary antibodies and ECL detection.

### ATP measurement

To measure the intracellular ATP level, SGC-7901 cells were incubated without (control) and with (40, 60 and 80 μM) Magnolol for 48 h. ATP level was measured using the ATP bioluminescence assay kit HSII (Roche Diagnostic, Indiannapolis, IN, USA) according to the manufacturer’s instructions.

### Acridine orange staining

Staining of cells with acridine orange was performed according to published procedure (38). In brief, cells were incubated without (control) and with Magnolol (40, 60, and 80 μM) and with rapamycin (positive control group) for 48 h and then acridine orange at a final concentration of 1mg/ml was added to cells for a period of 20 min in the dark at 37°C. Then, cells were washed twice with PBS. Images of cells were obtained under fluorescence microscopy.

### Flow cytometric quantification of acidic vesicular organelles (AVOs)

AVOs formation (autophagosomes and autolysosomes) is a characteristic feature of autophagy (39). For quantification of AVOs, we used flow cytometry after cells were stained by AO (40). AO is a weak base that accumulates in acidic spaces and gives bright red fluorescence [punctate staining (dots)] in the cytoplasm is detected by fluorescent microscopy. The intensity of the red fluorescence is proportional to the degree of acidity. Thus, the formation of AVOs can be quantified. Briefly SGC-7901 cells were harvested after treatment of 40, 60, and 80 μM of Magnolol for 48 h. Cell pellet was collected in an Eppendorf tube and cells were resuspended in 1 ml PBS. The staining of cells was done with AO (1 mg/ml) for 15–20 min in the dark at 37°C. Cells were centrifuged at 1000 rpm for 5 min; cells pellet was rinsed twice with PBS, and then resuspended in 400 μl PBS and analyzed on a flow cytometry using CellQuest software.

### PI staining assay

Cell death was measured by PI staining as previously described (41). Briefly SGC-7901 cells were trypsinized after the treating cells with 40, 60, and 80 μM of Magnolol for 48 h in the presence or absence of 3-MA, collected and resuspended with 1 ml PBS. Cells were stained with 0.5 ml of staining solution (40 mg ml^−1^ PI, 100 mg ml^−1^ RNaseA, 0.2% Triton-100) and cells were incubated in 37°C for 30 min in the dark. Cell death was measured by flow cytometry.

### Statistical analysis

For the statistical analysis of data, comparison between results from different groups were analysed with SPSS for Window Version 15.0. The Student’s t-test was employed to determine the statistical significance of the difference between different experimental groups and control group at P<0.05 value being regarded as statistically significant. All experiments were repeated at least three times. Data are presented as means ± standard deviation (SD).

## Results and Discussion

**Figure 1:**
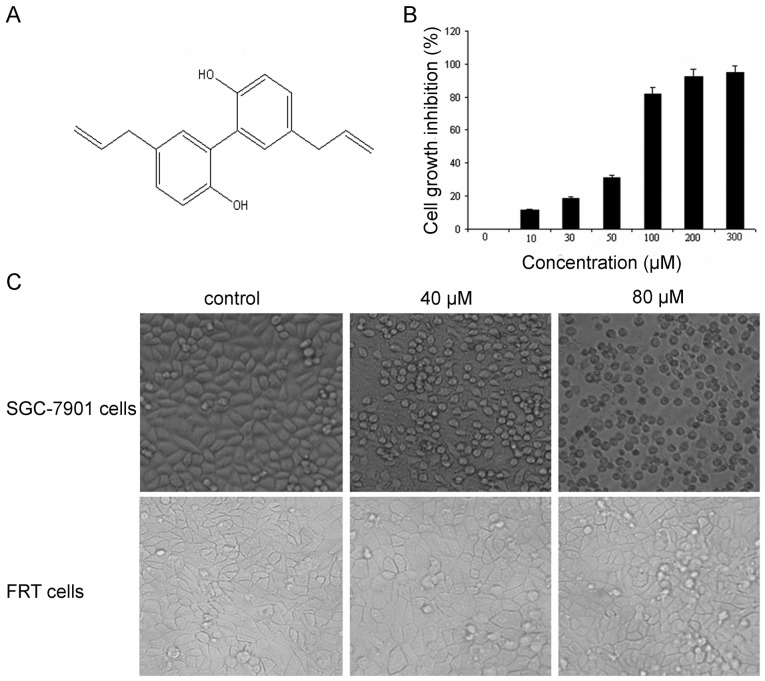
Effects of Magnolol on morphological characteristics and viability of SGC-7901 cells. (A) Chemical structure of Magnolol. (B) SGC-7901 cells were treated with various concentrations of Magnolol for 48 h. Cell death was measured by using MTT assay. Data shown are means ± SD (n=3). (C) Morphological changes of SGC-7901 and FRT cells were observed under the phase-contrast microscopy after treating without (control) and with 40 and 80 μM of Magnolol for 48 h.

We started the investigation with screening of natural compounds against human gastric adenocarcinoma SGC-7901 cells to evaluate the potential chemopreventive effects of natural compounds. Magnolol was one of them which showed antiproliferative effects against gastric adenocarcinoma SGC-7901 cells. The structure of Magnolol is shown in Fig. 1A. SGC-7901 cells were treated with different concentrations of Magnolol (0, 10, 30, 50, 100, 200 and 300 μM) for 48 h. Cell viability was measured by MTT assay and it was observed that Magnolol increased cell growth inhibition in a dose-dependent manner (Fig. 1B). Furthermore, the cytotoxicity of Magnolol was assessed by observing morphological changes in SGC-7901 cells under phase-contrasted microscopy and MTT assay. Phase contrast microscopic analysis of cell morphology was done following the exposure to Magnolol. Exposure of cells to 40, 60, and 80 μM of Magnolol for 48 h resulted in the significant decrease in cells number as compared to that of the control group. In addition, Magnolol exposure induced changes in shape and size of the cells and they turned round and shrunk while cells in the control group remained polygonal. The parallel treatment with Magnolol in normal FRT cells showed lesser effect (Fig. 1C). These results suggest that Magnolol can act as growth inhibitor of gastric adenocarcinoma SGC-7901 cells in a similar fashion as described in previous studies dealing with various other types of cancer cells including melanoma cells (21), colon cancer cells (24,25), prostrate cancer cells (26,27), human glioblastoma cancer cells (28,29), liver cancer cells (32), lung cancer cells (30,31), leukemic cells (14), thyroid carcinoma cells (33), and human urinary bladder cancer 5637 cells (35).

Cell cycle arrest and apoptosis are among several effective mechanisms involved in the induction of cell death (42). It is now well established that various checkpoints are involved in the proper progression of cell cycle in normal cells while in cancer cells regulation of cell cycle is altered due to abnormal cell growth. There are various factors such as DNA damage, exogenous stress signals, and defects during the DNA replication or failure of chromosomes to attach with the mitotic spindle which may disturb the normal functioning of these checkpoints and the loss of this regulation is the hallmark of cancer (43). To gain further insight into the mechanism of induction of cytotoxic effects of Magnolol on SGC-7901 cells, we then investigated the involvement of Magnolol in cell cycle arrest.

**Figure 2:**
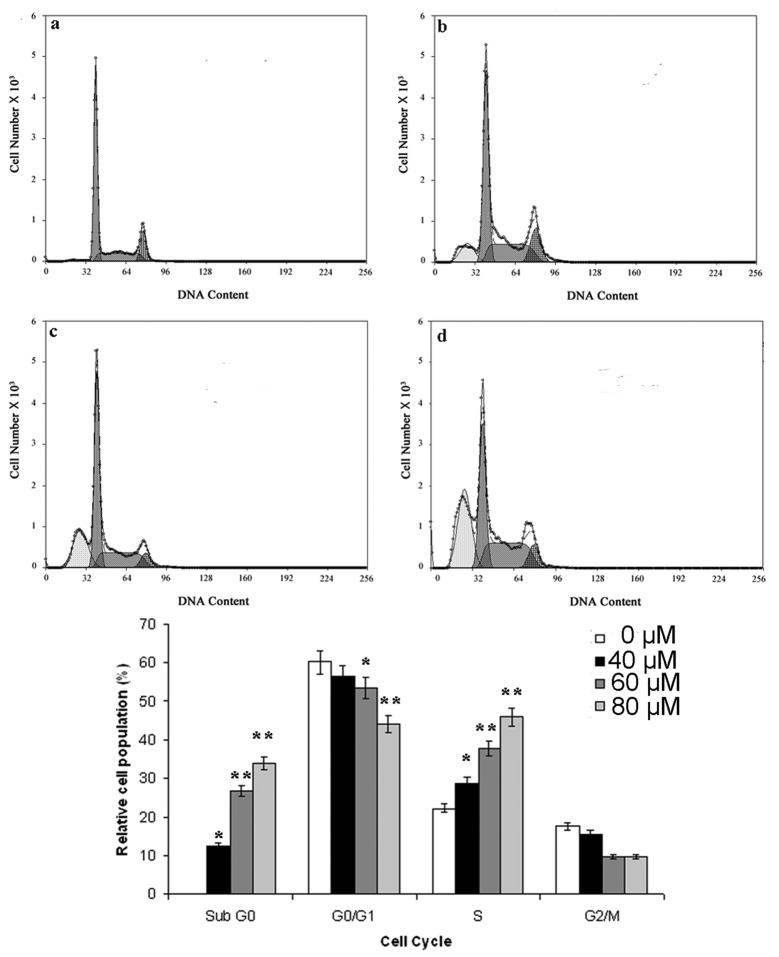
Effect of Magnolol on cell cycle distribution. SGC-7901 cells were treated with: (a) 0 μM, (b) 40 μM, (c) 60 μM, and (d) 80 μM of Magnolol for 48 h and then they were stained with PI for flow cytometric analysis. Histograms show number of cells/channel (y-axis) vs. DNA content (x-axis). The values indicate the percentage of cells in the indicated phases of the cell cycle. The data shown are representative of three independent experiments with the similar results. ^*^p<0.05; and ^**^p<0.01 compared with the control.

Previous studies have documented that Magnolol can arrest the cell cycle in different types of cancer cells (28,35). To decipher the effect of Magnolol on cell cycle progression of SGC-7901 cells, we treated the cells with 40, 60 and 80 μM of Magnolol for 48 h. The results showed that Magnolol arrested the cell cycle at S-phase, which supported previous results (30). The percentage of accumulation of the cells in the S-phase was increased from 22.27% in control group to 28.84, 37.84 and 45.99% in the cells treated with 40, 60 and 80 μM of Magnolol, respectively for 48 h (Fig. 2). In several settings, maintenance of proper cell cycle progression in cancer cells sets the stage for potential and effective treatment of cancer (44–46). These data strongly implicate S-phase cell cycle arrest by Magnolol as one of the mechanisms that induced cytotoxicity in SGC-7901 cells. Accumulated data indicate that many of the chemotherapeutic and chemopreventive agents have potential anti-proliferative effects via arresting the cell division at certain checkpoints in the cell cycle (47,48).

Apoptosis is one of the modes of cell death and induction of apoptosis is the key characteristic of anticancer drugs as it plays an imperative role in the elimination of damaged cells and the maintenance of homeostasis and many of the natural chemopreventive agents, including Magnolol, exert their effect via induction of apoptosis in cancer cells (21,49,50). To elucidate the effect of Magnolol on induction of apoptosis in SGC-7901 cells, these cells were treated with Magnolol and incubated with PI, analysed by flow cytometry and resulted in the increase of subG1 phase which represents the apoptotic cell population. The percentages of apoptotic SGC-7901 cells were 11.29, 24.72 and 31.76% after treating the cells with 40, 60 and 80 μM of Magnolol respectively for 48 h (Fig. 2).

**Figure 3:**
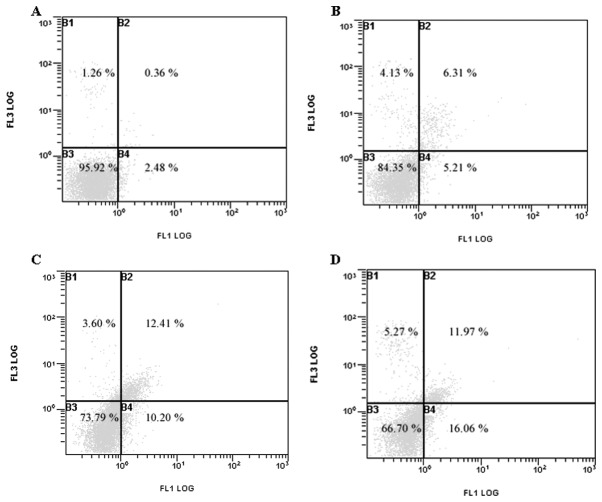
Apoptosis induced by Magnolol in SGC-7901 cells. SGC-7901 cells were treated with (A) 0, (B) 40, (C) 60 and (D) 80 μM of Magnolol for 48 h. Then they were stained with FITC-conjugated Annexin V and PI for flow cytometric analysis. The flow cytometry profile represents Annexin V-FITC staining in x-axis and PI in y-axis. The number represents the percentages of apoptotic cells in each condition. As shown, the cell populations in the lower right (Annexin V^+^/PI^−^) represents early apoptotic cells, upper right (Annexin V^+^/PI^+^) represents late apoptotic cells.

Furthermore, in order to gain confirmation of the apoptosis and to distinguish between early and late apoptosis, we performed flow cytometric analysis of apoptosis using annexin V-FITC and PI double staining. The results showed that apoptotic rates were 11.52, 22.61, and 28.03% after treating the SGC-7901 cells with 40, 60 and 80 μM of Magnolol respectively for 48 h (Fig. 3). Previous studies revealed that Magnolol inhibited the growth of the tumor cell proliferation by inducing apoptosis in various kinds of cancer cells such as melanoma cells (21), colon cancer cells (24,25), prostrate cancer cells (27), lung cancer cells (31), thyroid carcinoma cells (33), and human urinary bladder cancer 5637 cells (35). So results of present study were consistent with previously reported results.

**Figure 4:**
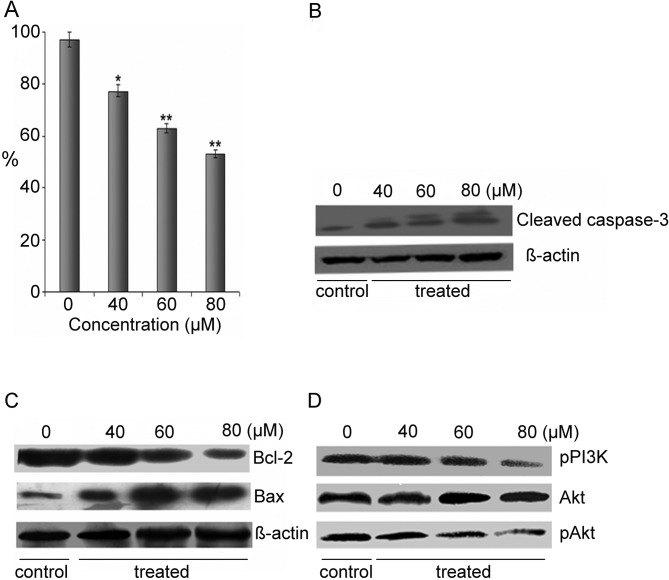
The effects of Magnolol on mitochondrial transmembrane potential and expression levels of apoptosis-related proteins. (A) The values indicate the percentage of Rho-123 fluorescence in the SGC-7901 cells treated without (control) and with (40, 60, and 80 μM) Magnolol for 24 h. The data shown are representative of three independent experiments with the similar results. ^*^p<0.05 and ^**^p<0.01 compared with the control. (B-D) Expression levels of Caspase-3, Bcl-2, Bax, pPI3K, pAkt, and Akt in SGC-7901 cells treated without (control) and with Magnolol (40, 60, and 80 μM) for 48 h were monitored by Western blot assay. β-actin was used as loading control. Western blots are representative of three independent experiments.

Apoptosis may be triggered either through the stimulation of death receptors located on the plasma membrane (extrinsic pathway) and/or within cells (intrinsic pathway) (49). Mitochondria are important organelles which are involved to release of apoptotic signals during an intrinsic pathway for the execution of apoptosis (51). Dysfunction of mitochondria leads to the dissipation of mitochondrial transmembrane potential and subsequently release of cytochrome *c* from the mitochondria into the cytosol. It is one of the mechanisms of caspase activation in a mainly apoptotic cell death (52). To determine the effects of Magnolol on mitochondrial transmembrane potential, SGC-7901 cells were incubated with 40, 60 and 80 μM of Magnolol for 24 h. The mitochondrial transmembrane potential was detected with Rho-123 staining in flow cytometry (Fig. 4A). This assay is based on the principle that decline in the fluorescence of Rho-123 was directly proportional to the decrease in the mitochondrial transmembrane potential (53). This is also in line with the results reported with Magnolol-induced apoptosis in human melanoma A375-S2 cells (21), human hepatoma (Hep G2) and colon cancer (COLO 205) cells (24), lung squamous carcinoma CH27 cells (31), and CGTH W-2 thyroid carcinoma cells (33).

Caspases play a central role in the apoptosis and caspase-3 is a frequently activated death protease, catalyzing the specific cleavage of many key cellular proteins (54). We examined the effect of Magnolol treatment on the activation of caspase-3 by Western blotting. As shown in Fig. 4B, Magnolol treatment led to a dose-dependent activation of caspase-3, where its cleavage was evident upon increasing the concentrations of Magnolol treatment for 48 h. Similar results were reported in human melanoma A375-S2 cells (21), human hepatoma (Hep G2), colon cancer (COLO 205) cells (24), human lung squamous carcinoma CH27 cells (31), and CGTH W-2 thyroid carcinoma cells (33) in which the caspase-3 is activated as result of treatment with Magnolol. Together our results and previously reported studies demonstrate that Magnolol-induced apoptosis involved the activation of caspase-3.

Our findings showed that Magnolol induced the apoptotic cell death associated with activation and cleavage of caspase-3 in SGC-7901 cells. Next we demonstrated the effect of Magnolol on Bcl-2 family proteins, including the anti-apoptotic (e.g., Bcl-2) and pro-apoptotic proteins (e.g., Bax). The balance between these two groups could profoundly affect cellular response to undergo apoptosis or not (55). This interaction ablates pro-survival function and activates the Bax and Bak, those render the cells to undergo apoptosis by permeabilizing the mitochondrial outer membrane (56,57). To reveal the effect of Magnolol on expression of Bcl-2 and Bax, Western blotting was performed. It was observed that Magnolol was involved in the up-regulation of Bax and down-regulation of Bcl-2 in a dose-dependent manner (Fig. 4C). These results were similar with previously reported studies in the other types of cancer cells such as lung squamous carcinoma CH27 cells (31) and human malignant melanoma A375-S2 cells (21).

Phosphatidylinositol 3-kinase/Akt is an important intracellular pathway that is frequently overexpressed in a wide variety of epithelial malignancies (58). Overexpression of Akt is associated with poor prognosis, tumor progression and resistance to systematic therapy in many types of human cancer including gastric tumors (59,60). Phosphorylation of Akt was able to significantly up-regulate the expression of Bcl-2, and down-regulate the expression of Bax in gastric cancer (58). Phosphatidylinositol 3-kinase/Akt pathway is a potential target of most of anticancer agents. Many researchers have focused on the PI3K/Akt pathway as potential target for therapeutic strategy against cancer (41,60). Next we evaluated the effects of Magnolol on the PI3K/Akt pathway by measuring the phospho-PI3K, total Akt, and sequential level of phospho-Akt proteins. It was observed that Magnolol markedly down-regulated the expression of phospho-Akt and PI3K in a dose-dependent manner after 48 h while total Akt protein levels remained constant during all treatments (Fig. 4D). In agreement to our other results, Magnolol induced a concentration-dependent down-regulation of phosporylated PI3K and Akt as reported previously (27,33).

**Figure 5:**
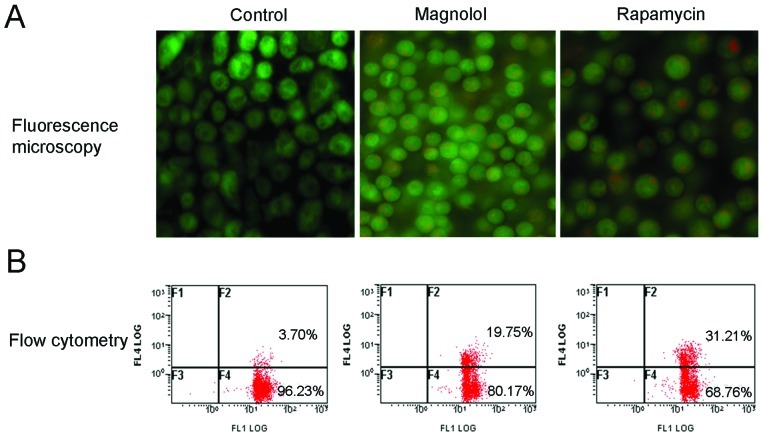
Formation of acidic vesicular organelles (AVOs) was observed by fluorescence microscopy and quantified by flow cytometry using the AO staining. (A) Cells were treated with Magnolol for 48 h before stained with acridine orange. Cells were examined by fluorescence microscopy. Representative images of cells from three independent experiments are shown. (B) The number represents the percentage of AVOs formation in SGC-7901 cells in each profile after treating cells without (control) and with Magnolol (80 μM) and rapamycin (positive control group) for 48 h. Three independent experiments were performed.

It has been reported that Magnolol induced autophagy in human lung cancer cells (30). Therefore, we hypothesized that Magnolol may induce autophagy in gastric adenocarcinoma SGC-7901 cells. To test our hypothesis, autophagy was analyzed by AO staining as described in Materials and methods. The formation of acidic vesicular organelles (AVOs) is one of the characteristic features of cells which passed through process of autophagy after exposure of different autophagy inducer agents (61,62). Autophagic vacuoles (AV) or autophagosomes are formed as result of sequestering parts of the cytoplasm or entire organelles, respectively, during the process of autophagy (63). We observed the effect of Magnolol treatment on the formation of AVOs in SGC-7901 cells using fluorescence microscopy upon staining with the lysosomotropic agent, acridine orange (AO). In fact, AO is a weak base that passes freely across the plasma membrane in a neutral state distinguished by green fluorescence. After entrance into acidic compartments, AO changed into protonated form which is distinguished by bright red fluorescence while control cells shown green fluorescence (Fig. 5A).

**Figure 6:**
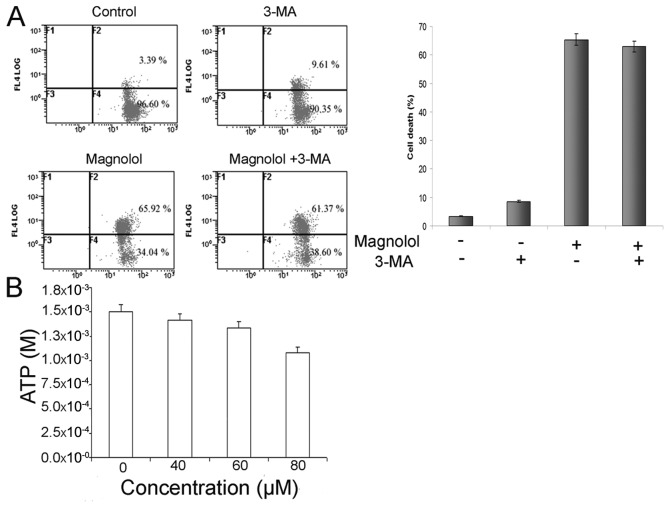
Effect of Magnolol-induced autophagy on cell death of SGC-7901 cells. (A) Cells were treated with Magnolol (80 μM) in the presence or absence of 3-MA. The number represents the percentage of dead cells in each profile after treating cells with 3-MA alone, Magnolol (80 μM) alone, Magnolol (80 μM) together with 3-MA with negative control group for 48 h. Three independent experiments were done. (B) Effect of Magnolol on intracellular ATP. Cells were treated with 40, 60 and 80 μM Magnolol for 48 h before the measurement of ATP by luminometric assay. Results shown are means ± SEM from three independent experiments.

For further verification of autophagy, we quantified AVOs, by flow cytometry using AO solution as described in Materials and methods. It was observed that there was no significant formation of AVOs at low concentration while AVOs were formed at high concentration of Magnolol treated cells as compared to normal cells (30) and rapamycin was used as positive control (Fig. 5B). Currently autophagic cell death has been studied as a potential method for cancer therapy. To determine the role of Magnolol-induced autophagy in cell death of SGC-7901 cells, we added the autophagy inhibitor, 3-methyladenine (3-MA) which controlled the autophagy pathway at various points (64). Initially effects of 3-methyladenine (3-MA) on cell growth inhibition were assessed. The viability of cells was more than 90% when they were treated with 3-methyladenine (3-MA) alone. Next SGC-7901 cells were treated with 80 μM of Magnolol and autophagy inhibitor 3-methyladenine (3-MA) together, cells were analyzed for cell death by flow cytometry using PI staining assay. Cells stained with propidium iodide were considered as dead cells (41). It was found that Magnolol-induced cell death was not suppressed when the cells were treated in combination with 3-MA (Fig. 6A). These results showed that Magnolol-induced autophagy is not involved in the induction of SGC-7901 cell death. It has been documented that autophagy may act as enabler of apoptosis, contributing in certain morphological and cellular events (ATP, cells blebbing and DNA fragmentation) that take place in apoptotic cell death, without leading to cell death by itself (64). We assumed that Magnolol-induced apoptosis may be involved in the ATP alteration. To verify this assumption, depletion of cellular ATP was observed after exposure of various concentrations of Magnolol (Fig. 6B). These results indicated that Magnolol-induced autophagy may affect the ATP level in SGC-7901 cells and supported observations which showed that autophagy may alter the morphological and cellular events that take place in apoptotic cell death (64).

**Figure 7:**
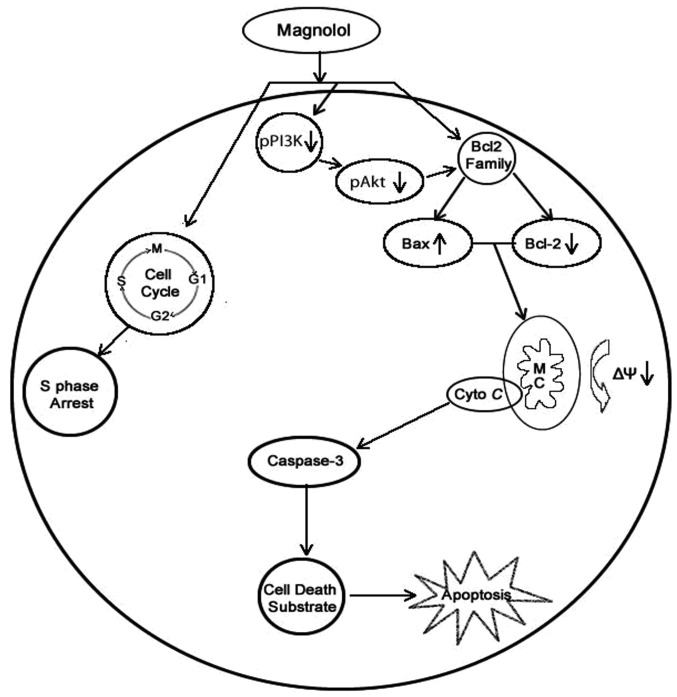
Hypothetical model of cytotoxic mechanism of Magnolol in human gastric adenocarcinoma SGC-7901 cells.

In conclusion, Magnolol-induced cell death of SGC-7901 gastric cancer cells via induction of apoptosis as well as S-phase cell cycle arrest. Analysis of apoptosis-related proteins in SGC-7901 cells revealed that Magnolol triggered the mitochondria-mediated apoptosis pathway as shown by increased ratio of Bax/Bcl-2, which led to dissipation of mitochondrial membrane potential (ΔΨm), sequential activation of caspase-3, and inhibition of PI3K/Akt (Fig. 7). Magnolol also induced autophagy at higher concentration but it is not involved in cell death of SGC-7901 cells. Magnolol-induced autophagy may alter the morphological and cellular events that take place in apoptotic cell death. But the exact role of Magnolol-induced autophagy in apoptosis is still unclear and needs further research. Taken together, these results suggest that Magnolol is a promising natural compound for the treatment of human gastric cancer and represents a potential candidate for *in vivo* studies of mono-therapies as well as combined anti-tumor therapies.
